# An interview with Nigel Harradine

**DOI:** 10.1590/2176-9451.19.4.030-037.int

**Published:** 2014

**Authors:** Mauricio Accorsi, Ricardo Moresca, John Pobanz, Weber Ursi

**Affiliations:** » Specialist in Orthodontics and Facial Orthopedics, Federal University of Paraná (UFPR). » MSc in Orthodontics, School of Dentistry - University of São Paulo (FOUSP).; » Specialist in Orthodontics and Facial Orthopedics, Federal University of Paraná (UFPR). » MSc in Orthodontics, Methodist University of São Paulo (UMESP). » PhD in Orthodontics, School of Dentistry - University of São Paulo (FOUSP). » Head of the Postgraduate program in Orthodontics, Positivo University (UP).; » Diplomate of the American Board of Orthodontics; » DDS and international speaker, Ogden, Utah, USA.; » MSc and PhD in Orthodontics, School of Dentistry - University of São Paulo/Bauru (USP-Bauru); » Full Professor, State University of São Paulo (UNESP); » Head of the Postgraduate program in Orthodontics, Association of Dental Surgeons/São José dos Campos/ São Paulo (APCD).

**Figure:**
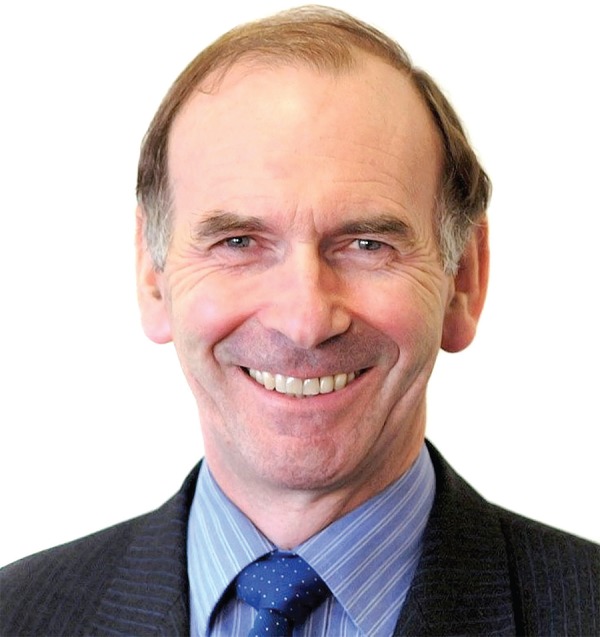


Dr. Nigel Harradine qualified as a dental surgeon from Guys Hospital, London and also underwent undergraduate medical training, qualifying again from Guys. A medical house physician post followed at Guys and then a year as an intern in London, Ontario before he settled on orthodontics as his chosen specialty and returned to the United Kingdom. A year of full-time general dental practice was followed by the two-year orthodontic course at the Royal Dental Hospital, London and Kingston Hospital, Surrey where he studied under Prof. Bill Houston and Harry Orton the Consultant orthodontist. In 1981, Dr. Nigel went to the Eastman Dental Hospital, initially as a lecturer and then as a senior lecturer in orthodontics. Since 1984 he has been a consultant at Bristol Dental Hospital and School where he is in charge of the undergraduate program and fully involved in the postgraduate teaching. Dr. Nigel has published innumerous papers, related to self-ligating brackets, TADs, orthognathic surgery, functional appliances, including a random prospective trial of the effects of third molar extraction on later incisor crowding. He has also contributed invited chapters to a number of textbooks. With David Birnie, Nigel Harradine has co-presented the annual Excellence in Orthodontics Course since 1987 and has lectured extensively throughout the United Kingdom including multiple presentations to the British Orthodontic Conference and the Consultant Orthodontists Group Symposium, including the Chapman Lecture, the Ballard Memorial Lecture, the Northcroft Lecture and the Webb Johnson Lecture. In total, Nigel has given 390 external invited presentations on the UK. In addition to Excellence in Orthodontics courses overseas, Nigel has delivered invited lectures and courses in Australia, France, Germany, Israel, Mexico, Malaysia, New Zealand, Norway, Serbia, South Africa, Switzerland, The Netherlands and the USA, where he has given six presentations to the Annual Session of the AAO, a two day course at Chapel Hill and keynote presentations to the Pacific Coast Society and in 2010 to the College of Diplomates of the American Board of Orthodontics - a total of 170 presentations. Away from the chairside and the podium, Nigel's roles have included being the President of the British Dental Association hospitals group, Chairman of the British Orthodontic Society scholarship committee, Chairman of the English Royal College working party on Read Coding, Secretary for seven years of the Royal College Orthodontic Audit working party, Secretary of the British Orthodontic Conference Organizing Committee, Chairman of the British Orthodontic Society Computer Users Group and a member of the British Orthodontic Society Council. He is a past Chairman of the Consultant Orthodontists Group of the BOS and a previous Clinical Director of Bristol Dental Hospital. Nigel was the initiator and inaugural chairman of the British Orthodontic Society Foundation which supports research and teaching in Orthodontics. Dr. Nigel kindly agreed to granted this interview to Dental Press Journal, and very interesting questions were formulated by Dr. Ricardo Moresca, Weber Ursi and Jonh Pobanz and myself. The main issue was SLB systems and we hope you can enjoy the wonderful experience and knowledge from Dr. Nigel Harradine.

Mauricio Accorsi

## It is undisputed that self-ligating brackets have been massively advertised by their manufacturers in marketing campaigns, targeting the end consumer. How do you evaluate the relationship between science and economic interests? The marketing can be considered one of the problems that self-ligating brackets faces for its popularity? Ricardo Moresca

I agree that some of a number of the claims made on behalf of self-ligation are in my view counter-productive in terms of encouraging their adoption. I think there are certainly worthwhile advantages in the use of self-ligation, some of which are definite and established and some of which remain untested at this stage. However, some of the less likely claims made on their behalf diminish the credibility of the advantages that are there to be had.

However, to put this in perspective, I don't think that developers of any bracket or other orthodontic equipment can be too heavily criticized for putting forward some over-optimistic claims for their baby. Without this enthusiasm, many of our most valuable tools would not have been developed. In particular, manufacturers are reluctant to invest heavily in a new bracket and then see the development through to a good, reliable, practical product without the prospect that it might be a game-changer. Self-ligating brackets (SLBs) are not alone in this respect. Many developers of some excellent and highly useful functional appliances believe strongly that their appliance grows mandibles to a substantial degree, although the evidence does not support this. Self-ligating brackets have attracted particular attention and criticism for some over-enthusiastic claims partly because they have been so successful in sales terms. Incidentally, the sales success does indicate that many orthodontists do find that these brackets have advantages.

## It is well known currently from clinically and scientific evidence, that we may have difficulties in rotational control when the self-ligating systems are used. One solution that has been presented to overcome this problem is to use two wires simultaneously (0.014-in and 0.016-in on a 0.022-in system, for example) in order to fulfill the bracket slot and eliminate this deficiency. What is your opinion about this approach? Ricardo Moresca

I don't think there is good scientific evidence that rotational control is different with SLBs. This has not been investigated much and often only incidentally. The many studies of alignment capability have found that this is very similar for SLBs and conventionally-ligated brackets. In a recent split-mouth study^1^ of canine retraction on 0.018" stainless steel wires, self-ligating brackets were found to give better rotational control of the canine than conventionally ligated brackets. SLBs are also not liable to the well-documented effects of elastomeric decay which can cause loss of tooth control.

Not all SLBs are equally effective. The only bracket for which the use of twin wires is advocated is the Smartclip bracket. The reason for that advocacy is not related to rotational control. The reason is that the Smartclip bracket is the only SLB where you do not open the clip or slide to insert or remove the wire; you have to push and pull the wire through the closed spring mechanism. This causes an inherent conflict of design. If the spring clip is sufficiently soft that the wire can be removed easily and without causing pain, then it tends to permit spontaneous loss of ligation. Early Smartclip brackets had a springclip which was too stiff. Engagement and removal of thicker wires was very uncomfortable. This was documented in a random controlled trial (RCT)^2^ as well as being a widespread clinical finding. So they redesigned and softened the clip, but there is a limit to how much you can do this. So several clinicians advocated using two thin wires rather than one thicker (eg 0.014 x 0.025-in or 0.016 x 0.025-in NiTi) wire to try to get round this dilemma and give comfort plus better control. They also advocate restricting the use of stainless steel wires as much as possible for the same reason. This is a sensible if inconvenient and somewhat restricting policy to get round the limitations of that particular design which no other manufacturer of the more than 30 types of available SLBs has adopted. A good clinician can often get round inherent limitations of a bracket or archwire (as we do with conventional brackets) but that does not make it the most sensible way to go.

Some clinicians feel that they have more trouble getting maximum alignment with SLBs and this may be a fact for them although the RCTs have not so far shown any difference. There is no reason arising from the bracket itself why this should be the case but I suspect the following are the potential reasons for some people finding this. Firstly, the brackets are unfamiliar in shape and whenever clinicians swap to a different make of bracket they find it harder to position as well as the brackets they are used to. Secondly, many SLBs are slightly narrower brackets in order to keep forces lighter and this may increase the need for accurate placement. Lastly, because the archwire has to be fully engaged with a SLB, any imperfections in bracket placement may be more fully expressed than if the elastomeric permits an incomplete engagement right to the end of treatment. Many clinicians including myself find no problems in getting the teeth very aligned with SLBs and in fact it is the cases which I treat with conventional ligation in which I sometimes have to regain control of one or more teeth.

## In 2008, I attended your conference at the AAO Congress in Denver. That occasion you asserted that there was still no evidence that self-ligating brackets afforded faster treatments, but this could change in the future. After almost six years and after a lot of research, what is your current opinion over this topic? Ricardo Moresca

There is still no evidence from RCTs that SLBs result in faster treatment although some controlled case series have shown this. The logical deduction is that SLBs do not offer a large inherent advantage in this respect. However, all RCTs to date have used SLBs as though they conventional brackets. They have used the same archwires, same appointment intervals and same biomechanics as with their conventionally ligated brackets. This is an understandable first approach and it has the advantage that the only difference between the two groups is the bracket so any difference can be attributed to the bracket. However, my view would be that the there is very extensive research to show that SLBs give a very different proportion of applied force to the movements you want and they swallow up much less in the resistance to those desired movements and this difference should be exploited in the treatment mechanics to get the best from the brackets.

E.g. with thick wires:

Good studies by Thorstenson and Kusy^3,4,5^ have investigated aspects of this topic. They examined the effects of varying active tip (angulation) on the resistance to sliding (RS). They found that angulation beyond the angle at which the archwire first contacts the diagonally opposite corners of the bracket slot causes a similar rise in resistance to sliding of both self-ligated (Damon SL) and conventional brackets. However, at all degrees of tip, the Damon brackets produced significantly less resistance to sliding. At an angulation of 6 degrees, which they chose to be clinically realistic for an 0.018 x 0.025-in stainless steel wire in an 0.022" slot, the difference is 60 gm per bracket which is very probably of clinical significance Their second paper^4^ compared different self-ligating brackets for resistance to sliding with active angulations. It quantifies a little more closely the lower resistance to sliding with passive self-ligation and points out that low resistance to tooth movement can lead to unanticipated movement. The third paper^5^ examined the same factors with wires of different sizes and in the dry state. The increase in friction when larger wires deflect the clips in active self-ligating brackets is quantified and the scanning electron micrographs of the different brackets show very clearly the relationship between small and large wires and active clips and passive slides. A study by Matarese^6^ is yet another to show that irregularity of the teeth does not prevent self-ligating brackets (Damon 2 in this instance) from producing significantly less resistance to sliding.

The views expressed on this important point vary widely, even when the same research is being quoted. For example, Brauchli et al^7^ quote data from Thorstenson and Kusy^3^ to the effect that for an 0.018 x 0.025-in wire, "with angulations of 7° between the archwire and slot, more than 94% of the RS is caused by binding". This has been taken to imply that the contribution to RS from the lowered friction with self-ligation is irrelevant whenever archwire activation causes binding. In fact, it is of course the very low friction with self-ligating brackets that makes any binding component such a high percentage of the overall RS. If the friction is zero, the binding component will constitute 100% of the RS. The other way of accurately describing the same data is as I have done in the preceding paragraph - "At a realistic angulation of 6 degrees for an 0.018 x 0.025-in stainless steel wire, the difference is 60 gm per bracket which is very probably of clinical significance". Pliska et al^8^ have also investigated the relationship between tipping moments and RS. They too found that at high levels of tip, any reduction in friction is drowned by the high binding, but at lower, more clinically realistic levels of activation, a potentially significant reduction in RS was seen as expressed in grams for force. In their words, "At the higher moment levels (4000 g-mm), no significant reduction in RS was found between conventional (CL) and self-ligating (SL) brackets on both SS and NT archwires. At lower levels of applied moment (2000 g-mm), reductions in RS of 18% (42.7g) and 18% (38.5 g) were found between the CL bracket and the best performing SL bracket on NT and SS, respectively."

### Studies of net aligning force - thin aligning wires:

The aligning force remaining after the RS is deducted is the other side of the resistance-to-sliding coin and is an equally important way of exploring and expressing RS. Until recently this has been inadequately appreciated and investigated. Almost all studies had measured the force resisting an archwire sliding along a wire. Three recent and excellent papers have explored this the converse - namely the net aligning force remaining in an archwire in spite of the resistance to sliding - when a tooth is significantly misaligned. Baccetti et al^9^ measured this in relation to vertical displacement of a tooth. The rather novel and well-considered difference from many studies is that their Instron machine did not measure the force required to pull an archwire through the brackets, but measured the residual net aligning force on the misplaced tooth. They found that at low levels of misalignment there was little difference between ligation methods, but beyond a 3 mm vertical misalignment, with conventional ligation the aligning force available was very substantially reduced by conventional ligation. At 6 mm displacement there was no remaining aligning force with conventional ligation but 40 to 120 gm remaining for self-ligating brackets. A current study by another research group of alignment of irregular teeth Nucera et al^10^ have also found the same redistribution of applied forces when self-ligation is used. "The NT wires released significantly lower forces with conventional ligation" and "clinicians should couple SLB systems with an alignment wire that exerts lighter forces".

So, if you have much less resistance to sliding and more force left over to do what you want and you combine this with a ligation that won't permit the tooth to rotate off the wire then you can - and should - break some of the conventional 'rules'. You should treat the cases more like Begg cases. You apply lighter forces (because less force is lost through RS) and you start applying all the intra- and inter-arch mechanics (eg class II traction) that you normally delay until later because we are taught to avoid the adverse effects of applying such forces to light wires. But if the forces are lighter and there is less obstruction to movement and you can pull on a canine tooth for example without the ligation allowing undue rotation, then you can get much more done earlier in the treatment. So the RCT that needs doing is to test two groups with the different ligation, but both groups treated with mechanics adapted to take advantage of SLBs. Then we will know about efficiency of treatment.

## Together with Dr. Birnie, you hold a course called "Excellence in Orthodontics" every year in the UK. Please explain what the course covers and what level of participants attend such courses. Weber Ursi

Yes, indeed, this year was our 28^th^ annual London course and we also give the course overseas on request. This year we are giving it in Sarawak as part of the Asian Pacific Conference in October. The course is open to anybody who is keen to learn, although almost everyone is a specialist orthodontist or an orthodontic trainee. A significant number of people come every few years to update. I attach this years London timetable and you will see that we cover a wide range of topics which vary each year depending on what we feel is most relevant in terms of the latest evidence or developments in technique. The course combines a critical look at the literature with lots of clinical examples. For more information, have a look at the EinO website at http://www.excellenceinorthodontics.com

## How does your medical training influence the way you treat your Orthodontic patients? Weber Ursi

Interesting question. I think it happens to give me a broader perspective on the place of orthodontics in the overall provision of healthcare and its contribution to quality of life. You don't need to have trained and worked as a medical doctor to have that but it help and that area of research is becoming much more important to defend the role of orthodontics in healthcare as opposed to a beauty treatment such as hairdressing or botox therapy.

## What is your preference regarding self-ligating brackets, passive or active? What is your opinion of using active SLB in the anterior teeth for better torque control and passive SLB in the posterior teeth for less friction? Weber Ursi

I have written in detail about this in a number of papers and book chapters but in summary, I think the active vs passive question is much less important than other aspects of bracket design such as how easily they open and close, how robust the self-ligating mechanism is, how secure against inadvertent opening it is, whether the mechanism is susceptible to calculus formation, whether it gets in the way of placing elastomeric chain or wire underties, whether the mechanism loses performance during treatment (one well-known bracket has been shown to suffer from this^11^) and similar considerations.

» Friction: Friction is higher with most active SLBs than with most passive SLBs but my feeling would be that those differences are of less clinical significance than the factors I have mentioned above.

» Torque: There is sound evidence from a number of papers that the labial force from an active spring clip supplies a clinically insignificant contribution to torqueing force. Brauchli et al^7^ ^,12^ are probably as good papers as any on this.

So I don't think that any differences in relation to torque control or friction are likely to be large enough to justify using both in the same arch. I have used 15 types of SLB and I still treat some case with the postgrads with conventional ligation and I find no differences in torqueing control. The only proviso is that you must make sure the archwire is fully engaged behind the face of the slot for the torque force to be as it should for that size wire. With a passive SLB (or a molar tube), the wire has to be fully engaged or you cannot get the slide to close or the wire into the molar tube. With a wire or elastomeric ligature it is possible to get the rectangular wire incompletely into the slot and still send the patient away with all the teeth 'ligated'. As it happens, I am speaking about this topic this week at the AAO in New Orleans.

## Why do you think existing passive self ligation systems lack rotation and torque control during finishing? John Pobanz

I don't - see above. If someone thinks that a ligature would provide rotation or torque control, then why not add a ligature to the SLB? This can easily be done with almost all SLBs. I think they would be disappointed with the additional effect of the ligature and this indicates that the ligation is not the problem.

What is your protocol for posterior crossbite correction? John Pobanz

I usually find that cross elastics from the first visit with the bite propped open work well. I may prop the bite posteriorly with reinforced glass ionomer on the lower molars or anteriorly with bite turbos if the overbite needs reducing. People tend to wear elastics well at the start of treatment and the teeth tip quickly over the bite because the light round wires permit that. Once the teeth are not in crossbite, the occlusion helps upright the tipped teeth and thicker wire do the rest. If the crossbite is unusually severe and generalized, I sometimes use a quad helix. The nuisance of that is that you have to separate, fit bands, send it to the lab or adjust a pre-formed quad in the mouth and of course you cannot easily align the molars well until you remove the quad, so it has to be worth the extra hassle. All the literature supports the view that in the long term the difference in bodily /orthopedic change is small with archwires vs quads vs RME. A current study^13^ compares expansion with a quad helix and conventional ligation with archwire expansion using Damon 3MX brackets. The expansion achieved was the same in both groups (approximately 5 mm at the premolars and 3.5 mm at the molars) with slightly more buccal tipping in the Damon group. This can be interpreted as suggesting that:

» Quad helix appliances add little to the treatment.

» There is no "Frankel -like" effect of Damon brackets and buccal soft tissues.

This paper also quotes other research finding similar buccal tipping of the teeth with Damon and with RME so you can pursue that aspect via this current paper. I personally have not used RME for years. The CT research by Garib et al^14^ has demonstrated very large dehiscences of buccal bone following rapid maxillary expansion, so if you use RME it should be done cautiously and in young patients.

One other trick which works well as an alternative to a quad is to place an additional expanded thick wire in the headgear tubes at the start of treatment and ligate it to the main archwire at the front of the mouth to keep it in place and in control. This assumes that you have headgear tubes on your molars - which I usually don't.

## Do you see any merit to stainless steel mini-screws compared to titanium mini-screws? John Pobanz

No. Titanium is more biocompatible and will osseointegrate more but whether this makes any difference in miniscrew usage, I wonder.

## Orthodontics as specialty, seems to experiencing a paradigm shift without precedents, and SLBs are playing an important role in this context, along with 3D imaging (CAD/CAM Systems), TADs, Genetic Engineering, Bioengineering, Integrative Medicine, Minimally Invasive Dentistry and so on. All together to achieve better, predictable, safer and faster results. So, do you believe that "this is really the future"? in other words, do you believe that the contemporary Orthodontist has to relearn everything again? Mauricio Accorsi

I certainly think that the rate of development of knowledge and of new technologies is currently rapid and that we do really have to keep up with the advances in both of those areas. Firstly, these developments are improving our ability to consistently deliver good results with a better patient experience. For example I think that intraoral scanners have at last become good enough to be widely adopted. I think that in a few years alginate impressions will be a small part of our record collection and appliance manufacture. I think that CAD/CAM design of individually customized appliances will become much more widely used and that treatment will be shown to be better and quicker. Some of the less invasive technologies which are currently advocated for speeding up tooth movement may prove to be effective, which would also enhance the patient experience. The other effect of new technology in orthodontics is that the scope of diagnosis and treatment will probably widen. We have already seen this with the introduction of TADs. Malocclusions such as some anterior open bites and some class III can now be treated using TADs that previously required orthognathic surgery. This is an exciting and significant advance. Cone beam CT scanning expands our ability to ethically investigate the effects of orthodontics on airway dimension and our role in treating sleep apnoea. This is potentially important for a speciality which is liable to criticism that it is purely cosmetic with little health benefit. Orthodontic research tools are also important in that context, with the development of better ways of measuring the oral-facial quality of life now starting to demonstrate the wider effects of orthodontics on all-round well-being.

This does not mean that we have to unlearn anything we have learnt but it does mean that we have to progressively change our practice if we are to do what is best for our patients. This is not comfortable for many people. We get to be very good with the ideas we have and very skilful with the clinical tricks that we know and faced with a busy clinic we tend to stick with what we know we can do without the day being stressful. We go away from a conference and think that we have heard some very interesting things, but the urge to change our practice may get put on the back burner in the interests of not having the stress of being a beginner with something new. However we are always building on previous skill and ability to relate well to patients and to understand the underlying mechanisms of tooth movement and appliance design. That is a strength and change is interesting, stimulating and rewarding and should keep us enthusiastic and engaged.

## Reducing the number of extractions during orthodontic treatment has been one of the supposed advantages attributed to SLBs treatments. Do you think this can be true and, based on which factors do you make your extractions decision? Mauricio Accorsi

To summarize on that question, I think that self-ligation probably makes it easier to squeeze teeth into line if they are substantially irregular and crowded. We don't yet know for certain if they are indeed more efficient at doing that, because no RCT has yet focused on substantially crowded cases, but the laboratory evidence gives reason to propose that this may be the case and you will have seen some impressive individual cases. So if you wish to extract less in such cases, self-ligation may make that easier to achieve. However, that does not of itself mean that you should change your treatment goal in terms of arch width or incisor labio-lingual position. I personally do not believe that the way you align teeth alters the factors that determine where they will be stable or where they will look nice or where they will enable a good occlusion. We need to distinguish the means of aligning the teeth from the goals we have for their final position. There have also been some well-known hypotheses that the lower forces needed to move teeth with SLBs may enable the lips to more effectively resist labial movement of the incisors - the "lip bumper effect". I remain in need of convincing about that suggestion. If it does apply, I suspect it is only in those cases where the lips are tight and strong and the clinician is careful to keep the forces low that this effect might apply. So far, no studies have supported this effect, but to date, the most likely sample of patients and the best use of the demonstrated biomechanical differences of SLBs have not been used in a RCT. There has also been the proposal that tongue posture may be altered when the lip forces are able to interact with the forces we apply. I remain sceptical about that idea too, although it is difficult to test. Cases treated with SLBs which show less incisor proclination and more uprighting of buccal teeth than we might expect probably contain lessons for us about planning and treatment but not necessarily that we are altering tongue behavior or permitting the lips to compete with orthodontic forces.

With advances in appliances, extractions have become less necessary for us to achieve a good set of study models and that is good and useful and helps us to remove and throw away fewer healthy teeth, but the final position of that occlusion in relation to the face and the supporting tissues continues to be determined by our understanding and continually developing knowledge of aesthetics, occlusion and periodontal health.

You may be familiarized with Dr. Chris Chang's approach for Class III and gummy smile treatments by means of OBS (OrthoBoneScrews). What is your opinion about this philosophy? Mauricio Accorsi

Yes I am familiar with Chris Chang's approach to this (and to his very entertaining lecturing style). I have not tried his exact recipe but I have used Hugo de Clerck's miniplates for a number of young class 3 patients with some good success and some teething problems with getting the surgery done just 100% right. I have also used anterior midline miniscrews to intrude upper incisors. I think that this area of biomechanics offers a very exciting period of development in orthodontics. As with all new techniques, they are not as universally successful or without problems and a necessary learning curve as the enthusiastic promoters tend to suggest, but the benefits are definite and the scope of orthodontics is definitely increased.

## Figures and Tables

**Table:** 

Angulation (degrees)	Damon SL	Conventional bracket
0	0	34
3.5	0	55
6.0	80	140

Resistance to sliding (RS) for different bracket angulations with a 0.018 x 0.025-in archwire. Forces in cN. The difference is 60 gm at 6 degrees of angulation (Source: Thorstenson e Kusy^3,^ 2001).
